# Effects of rhaponticum carthamoides versus glycyrrhiza glabra and punica granatum extracts on metabolic syndrome signs in rats

**DOI:** 10.1186/1472-6882-14-33

**Published:** 2014-01-20

**Authors:** Michael Dushkin, Marina Khrapova, Gennadiy Kovshik, Marina Chasovskikh, Elena Menshchikova, Valeriy Trufakin, Anna Shurlygina, Evgeniy Vereschagin

**Affiliations:** 1 Laboratory of Molecular and Cellular Mechanisms of Therapeutic Diseases, Institute of Internal Medicine, Siberian Branch of the Russian Academy of Medical Sciences, Novosibirsk, Russia; 2 Institute of Physiology and Fundamental Medicine, Siberian Branch of the Russian Academy of Medical Sciences, Novosibirsk, Russia; 3 Center of Clinical and Experimental Medicine of Siberian Branch of the Russian Academy of Medical Science, Novosibirsk, Russia

**Keywords:** *Rhaponticum carthamoides*, Metabolic syndrome, Corticosterone, Inflammatory cytokines

## Abstract

**Background:**

*Rhaponticum cathamoides* (RC) is an endemic wild Siberian herb with marked medicinal properties that are still poorly understood. The aim of this study is to investigate the therapeutic potential of RC extract (ERC) compared to the effects of *Glycyrrhiza glabra* (EGG) and *Punica granatum* extracts (EPG) in a rat model with high-fat diet-(HFD)-induced signs of metabolic syndrome; therefore, this study addresses a significant global public health problem.

**Methods:**

Six-month-old male Wistar Albino Glaxo rats were subjected to eight weeks of a standard diet (SD), HFD, or HFD in which ERC, EGG, or EPG powders were incorporated at 300 mg/kg/day. The serum lipid profile, corticosterone and cytokine concentrations, glucose tolerance, systolic blood pressure, triacylglycerol accumulation, and PPARα DNA-binding activities in the liver samples were determined.

**Results:**

In contrast to EGG and EPG, an ERC supplement significantly reduced the weight of epididymal tissue (19.0%, p < 0.01) and basal serum glucose level (19.4%, p < 0.05). ERC improved glucose intolerance as well as dyslipidemia more efficiently than EGG and EPG. EGG but not ERC or EPG supplementation decreased systolic blood pressure by 12.0% (p < 0.05). All of the tested extracts reduced serum IL6 and corticosterone levels induced by HFD. However, the lowering effects of ERC consumption on the serum TNF-α level and its restoring effect on the adrenal corticosterone level significantly exceeded the improvements induced by EGG and EPG. ERC intake also reduced triacylglycerol accumulation and increased the PPARα DNA-binding activity in the liver more significantly than EGG and EPG.

**Conclusions:**

ERC powder supplementation improved glucose and lipid metabolism more significantly than EGG and EPG in rats fed on HFD, supporting the strategy of *R. carthamoides* use for safe relief of metabolic syndrome and its related disturbances such as inflammation, stress, and hepatic steatosis.

## Background

Metabolic syndrome, a condition defined by a cluster of cardiometabolic risk factors including visceral adiposity, diabetes mellitus, dyslipidemia, and high blood pressure represents a significant global public health problem [1]. Among environmental factors, the high-fat diet and sedentary lifestyle common in the Western world are considered to be the major causes of obesity-associated impaired glucose tolerance and dyslipidemia [2]. Metabolic syndrome is also often characterized by chronic inflammation and hepatic steatosis [3]. Early treatment of people with metabolic syndrome may prevent the development of cardiovascular disease. Current treatment strategies include lifestyle modifications with pharmacological interventions targeted at complex manifestations of the metabolic syndrome as necessary [4]. Multiple biological targets controlling these manifestations will require a simultaneous application of different classes of drugs such as statins, thiazolidinediones, fibrates, biguanides, sulfonylureas, antihypertensive drugs, and many others for the attainment of beneficial effect. Although significant progress has been made in the development of therapeutic strategies for reducing risk factors for cardiovascular disease [5-7], the integrated management of metabolic syndrome is often an elusive goal in practice. The problem of integrated management of metabolic syndrome arises due to the long-term use of expensive medications that sometimes may result in adverse side effects. Recently, a number of innovative nutritional strategies based on a long and successful practice have been proposed as a safe alternative treatments to reduce the morbidity as well as the cost of metabolic syndrome treatment [8-10].

To date, more than 1000 officinal plant treatments for metabolic disorders have been reported, although only a small number of these have received scientific and medical evaluation to assess their comparative efficacy [11]. Therefore, more screening trials of commercial herbal products are needed to develop functional as well as analytical bases for standardization of dietary supplements [12]. Among herbs of this kind, nutritional ingredients of *Glycyrrhiza glabra* and *Punica granatum* are widely used in Indian and Chinese traditional medicine. The antidiabetic and anti-obesity effects of *G. glabra*[13,14] and *P. granatum*[15,16] are relatively well studied in animal models. In particular, the licorice flavonoids have recently been shown to suppress abdominal fat accumulation and increase in blood glucose level in obese rats [17] and mice [18]. High total polyphenol content is related to antidiabetic and antioxidant effects of *Punica granatum* extracts observed in mice [19] and rats [20].

*Rhaponticum carthamoides* (Willd) Iljin, commonly known as maral root or Russian leuzea, has been widely used in the traditional Siberian medicine, mostly to treat overstrain and common weakness after illnesses, as a stimulant, and a remedy against male sex dysfunction. The principal bioactive constituents of this plant are ecdysteroids, flavonoids, and phenolic acids. The extracts and preparations from this plant, which are practically safe, exhibited various additional antioxidant, immunomodulatory, antitumor, and antimicrobial effects [21]. However, relatively scarce literature is available on the medicinal properties of *R. carthamoides* as a treatment for metabolic disorders. In addition, the effect of *R. carthamoides,* especially on the high fat diet-induced metabolic syndrome development, remains to be clarified. The potential of *R. carthamoides* in the inhibition of the basic manifestations of metabolic syndrome is not sufficently studied. In the present study, we tested the effects of the ethanolic extract of *R. carthamoides* root versus the commercially available ethanolic extracts of *G. glabra* root and *P. granatum* peel with proclaimed antidiabetic and anti-obesity properties on the signs of metabolic syndrome in an obese rat model.

## Methods

### Plant materials and chemicals

Commercial ethanol extract powder from the root of *R. carthamoides* (wild-growing) (20-hydroxyecdisone content of 2.2% was standardized by HPLC analysis), licorice root extract powder (glycyrrhizic acid (15.0%) was conditioned by HPLC analysis) and pomegranate peel extract powder (ellagic acid (40.0%) was conditioned by HPLC analysis) were obtained from KIT Co., Ltd. (Altai State Technical University, Barnaul, Russia), Wixi Cima Science Co., Ltd. (Jiangsu, China (Mainland)), and Xian Yuensun Biological Technology Co., Ltd. (Shaanxi, China (Mainland)), respectively, and were stored at +4°C until use. All other chemicals were analytical grade.

### Animals, diets, and experimental design

Male Wistar Albino Glaxo rats, six months old, were obtained from the Animal Center of the Institute of Cytology and Genetics (Novosibirsk, Russia) and were housed individually in cages in an air-conditioned room (24.2°C) with a 12 h light/dark cycle and food and water provided *ad libitum*. All animal experiments were performed according to the animal ethics guidelines of the European Communities Council Directive (86/609/EEC) and approved by the Animal Care Committee of the Institute of Internal Medicine, Novosibirsk, Russia. The animals were randomly split into 5 groups of 10 rats each. Group I was maintained on briquette feed standard diet (SD group) containing 10% fat-derived calories, while group II was fed a high-fat diet (HFD group) containing 60% fat-derived calories (Laboratorsnab, Moscow, Russia) for 8 weeks. The rats of groups III, IV, and V were fed an HFD supplemented with the powder extracts of *R. carthamoides* (ERC group), *G. glabra* (EGG group), and *P. granatum* (EPG group), respectively, at a daily dose of 300 mg/kg of body weight (b.w.) for 8 weeks. Animals were weighed once a week, and food intake was measured daily. At the end of each experimental period, rats were deprived of food for 16 h, then anesthetized with an intraperitoneal injection of sodium pentobarbital (50 mg/kg b.w.) and sacrificed. Epididymal adipose tissue, adrenal gland and liver were extracted and weighed.

### Intraperitoneal glucose tolerance test

At week 8 of the experiment, rats were deprived of food overnight (16 h) and after collecting a fasting blood sample from a tail vein were injected intraperitoneally with D-glucose (50% stock solution in saline, 5 g/kg b.w.). Blood glucose concentration was measured at 60, 120, and 180 min after injection by One Touch Horizon glucometer (Lifescan, Johnson and Johnson, NJ, USA).

### Blood pressure

At baseline and further weekly, systolic and diastolic blood pressure were measured by a noninvasive pressure device using volume pressure recording, CODA 2 (Kent Scientific, Torrington, CT, USA), on non-anesthetized rats restrained in the thermic plastic chamber.

### Determination of serum triacylglycerol, cholesterol, free fatty acid, corticosterone, and cytokine content

Blood samples were collected in tubes and centrifuged at 2400 g. Serum concentration of triacylglycerol (TG), total cholesterol (TC), and high-density cholesterol (HDL-C) were measured by an enzymatic colorimetric method using commercial enzyme assay kits (Olvex Diagnosticum, St. Petersburg, Russia). Low density lipoprotein cholesterol (LDL-C) content was calculated using the formula: LDL-C = TC - (HDL-C + TG/2.2). Serum concentration of free fatty acid (FFA) was measured by an enzymatic colorimetric assay according to the manufacturer^’^s protocol (free fatty acid, Half-micro test; Roche Diagnostics, Penzberg, Germany). Serum and adrenal homogenate corticosterone was assayed by enzyme-linked immunosorbent assays. Sensitivity limits were 30 pg/mL and coefficients of variation were 7-9% using the Enzyme Immunoassay (EIA) kit (Cayman Chemical, Ann Arbor, MI, USA). Serum tumor necrosis factor-α (TNF-α) and interleukin-6 (IL-6) levels were measured by commercially available rat ELISA kits (eBioscience, CA, USA).

### Determination of liver triacylglycerol content

Approximately 0.2 g of hepatic tissue was homogenized in 0.15 M NaCl solution and extracted using a hexane:isopropanol mixture (3:2, v/v), containing 0.005% (wt/vol) butylated hydroxytoluene, according to method of Hara and Radin [22]. After 10 min centrifugation at 3,000 g and 10°C, the upper organic phase was collected and evaporated under liquid nitrogen. Dry total lipids were resuspended in 10% Triton X-100 and isopropanol, and TG content was measured using a commercial enzyme assay kit (Olvex Diagnosticum, St. Petersburg, Russia) on an automatic biochemical analyzer.

### Preparation of liver nuclear protein extract

Nuclear protein was isolated according to the method of Kang et al. [23]. Liver tissue (0.5 g) was homogenized in a buffer containing 0.32 M sucrose, 10 mM Tris · HCl, pH 7.4, 1 mM EGTA, 2 mM EDTA, 5 mM NaN_3_, 10 mM β-mercaptoethanol, 20 μM leupeptin, 0.15 μM pepstatin A, 0.2 mM PMSF, 50 mM NaF, 1 mM sodium orthovanadate, and 0.4 nM microcystin. The homogenates were centrifuged (1,000 *g*, 10 min). The pellets were solubilized in Triton buffer (1% Triton X-100, 150 mM NaCl, 10 mM Tris·HCl, pH 7.4, 1 mM EGTA, 1 mM EDTA, 0.2 mM sodium orthovanadate, 20 μM leupeptin A, 0.2 mM PMSF). The lysates were centrifuged (15,000 *g*, 30 min, 4°C), and the supernatant (nuclear extract) was stored at -80°C until use. Protein concentrations were estimated using the Bio-Rad (Hercules, CA) DC protein assay.

### Determination of liver PPARα transcription factor activity

PPARα transcription factor activity in liver nuclear extracts was determined using ELISA-based Cayman Chemical PPAR-α transcription factor assay kit (Cayman Chemicals, Ann Arbor, MI, USA) that detects PPAR-α bound to PPAR response element-containing double-stranded DNA sequences. Nuclear extract protein sample (40–50 μg) was used for determination of PPARα activity using the protocol described in the product manual, and absorbance was measured at 450 nm.

### Statistical analysis

Results are expressed as the means ± standard error (S.E.M.). Statistical analysis was performed by applying one-way analysis of variance (ANOVA) followed by the Tukey post hoc test. Difference between groups was considered significant at p < 0.05.

## Results

### Effects on weight of epididymal adipose tissue

**Figure 1:**
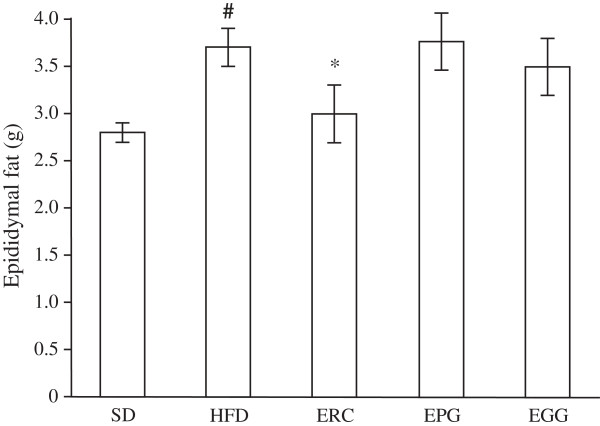
**Epididymal fat tissue weight of rats fed a standard diet (SD), a high-fat diet (HFD), and an HFD supplemented with *Rhaponticum cathamoides *extract (ERC), *Punica granatum *extract (EPG) and *Glycyrrhiza glabra *extract (EGG) (300 mg/kg per day) for 8 weeks.** Data represent the mean ± S.E.M. (n = 10). The statistical significance of differences between rats fed HFD and either SD (# p < 0.001) or HFD supplemented with ERC, EPG or EGG (*p < 0.01) was determined.

No significant differences in body weight and energy intake between the groups were observed (data not shown). Eight weeks after high-fat diet exposure, the weight of epididymal fat in HFD rats was significantly elevated (p < 0.001) compared to the control (SD) rats (Figure 1). The supplementation of ERC for eight weeks in the HFD rats significantly lowered the weight of epididymal fat (p < 0.05), whereas supplementation of EGG or EPG did not alter this characteristic.

### Effects on glucose tolerance test and blood lipids

**Figure 2:**
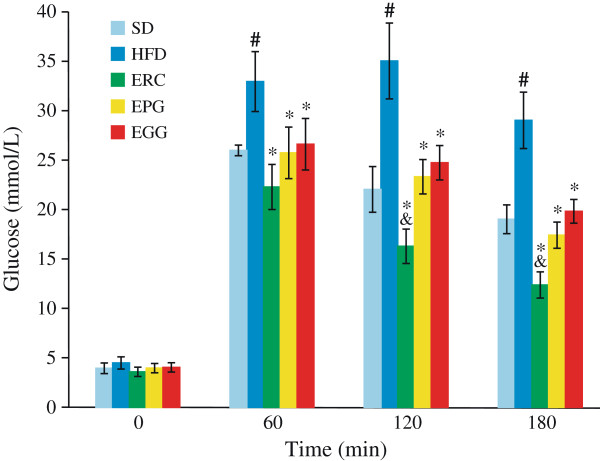
**Glucose tolerance test of rats fed standard diet (SD), high-fat diet (HFD), and HFD supplemented with *Rhaponticum cathamoides *extract (ERC), *Punica granatum *extract (EPG) and *Glycyrrhiza glabra *extract (EGG) (300 mg/kg per day) for 8 weeks.** Data represent the mean ± S.E.M. (n = 10). The statistical significance of differences between rats fed HFD and either SD (#p < 0.001) or HFD supplemented with ERC, EPG or EGG (*p < 0.01), between rats fed HFD supplemented with ERC and either EPG or EGG (& p < 0.05) was determined.

Serum glucose value was increased by 20.0% (p < 0.05) in HFD rats compared to SD rats (from 4.46 ± 0.16 to 5.34 ± 0.32 mmol/L). The supplementation of ERC, but not EGG or EPG, significantly reduced basal glucose levels by 19.4% compared to the HFD group (p < 0.05; data not shown). An intraperitoneal glucose tolerance test was performed at the end of week eight of the experiment. The HFD group serum glucose levels were significantly higher at 60, 120 and 180 min after glucose loading compared to the SD group, and all of the three plant extracts studied significantly reversed this effect at 60 min and at every time point thereafter (Figure 2). At the same time, Figure 2 demonstrates that serum glucose levels were significantly lower in rats receiving ERC (p < 0.05), than in rats receiving EGG or EPG at 120 and 180 min after glucose loading.

As shown in Table 1, the HFD significantly increased the serum levels of TC (p < 0.001), TG (p < 0.001), LDL-C (p < 0.001), and FFA (p < 0.001) compared to SD, whereas HDL-C serum content decreased (p < 0.05). The ERC intake markedly reduced the serum TG (by 22.6%, p < 0.05), TC (by 18.0%, p < 0.05), LDL-C (by 52.0%, p < 0.01), and FFA levels (by 32.4%, p < 0.05) and restored HDL-C level (by 37.0%, p < 0.05), indicating that ERC reversed the dyslipidemia induced by HFD (Table 1). Some improvement in lipid profiles was also noted for EGG (significant decrease of TG and FFA content by 25.0 and 27.0%, respectively) and EPG (significant decrease of LDL-C level by 26.0%) groups compared to HFD group. The other lipid indicators in the EGG and EPG groups demonstrated a weak tendency to recovery, albeit not significantly (p > 0.05).

**Table 1:** **Serum lipids content (mmol/L) in rats fed standard diet (SD), high-fat diet (HFD), and HFD supplemented with *Rhaponticum cathamoides *** **extract (ERC), *Punica granatum *** **extract (EPG) and *Glycyrrhiza glabra *** **extract (EGG) (300 mg/kg per day) for 8 weeks**

**Lipid**	**SD**	**HFD**	**HFD + ERC**	**HD + EGG**	**HFD + EPG**
TC	2.03 ± 0.21	2.82 ± 0.24^#^	2.31 ± 0.19*	2.89 ± 0.32	2.59 ± 0.22
LDL-C	0.47 ± 0.25	1.46 ± 0.35^#^	0.70 ± 0.24*	1.22 ± 0.34	1.08 ± 0.28*
HDL-C	1.20 ± 0.11	0.81 ± 0.21^#^	1.11 ± 0.14*	1.08 ± 0.24	0.95 ± 0.22
TG	1.79 ± 0.14	3.27 ± 0.37^#^	2.53 ± 0.25*	2.45 ± 0.35*	2.82 ± 0.37
FFA	0.76 ± 0.09	1.43 ± 0.24^#^	1.11 ± 0.12*	1.04 ± 0.12*	1.39 ± 0.16

Data represent the mean ± S.E.M. (n = 10). The statistical significance of differences between rats fed HFD and either SD (#p < 0.01) or HFD supplemented with ERC, EPG or EGG (*p < 0.05) was determined.

### Effects on systolic blood pressure

**Figure 3:**
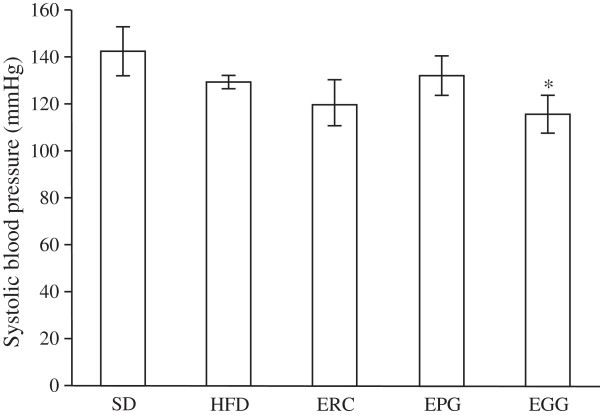
**Systolic blood pressure of rats fed a standard diet (SD), a high-fat diet (HFD), and HFD supplemented with *Rhaponticum cathamoides *extract (ERC), *Punica granatum *extract (EPG) and *Glycyrrhiza glabra *extract (EGG) (300 mg/kg per day) for 8 weeks.** Data represent the mean ± S.E.M. (n = 10). The statistical significance of differences between rats fed HFD and either SD or HFD supplemented with ERC, EPG or EGG (*p < 0.05) was determined.

No significant differences (p>0.05) were observed in systolic blood pressure of SD and HFD groups. Interestingly, HFD resulted in a tendency to a decreased systolic blood pressure at the end of week eight (Figure 3), albeit not significantly (p>0.05). The supplementation of EGG resulted in a significant decrease of systolic blood pressure by 12.0% (p < 0.05) compared to the HFD group. As shown in Figure 3, no significant difference was observed upon the supplementation of ERC or EPG in HFD compared to HFD group.

### Effects on serum and adrenal corticosterone

**Figure 4:**
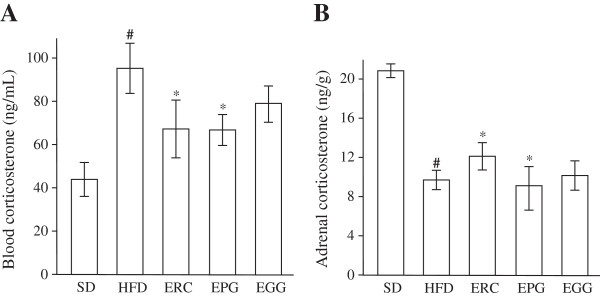
**Concentration of serum (A) and adrenal (B) corticosterone of rats fed standard diet (SD), high-fat diet (HFD), and HFD supplemented with *Rhaponticum cathamoides *extract (ERC), *Punica granatum *extract (EPG) and *Glycyrrhiza glabra *extract (EGG) (300 mg/kg per day) for 8 weeks.** Data represent the mean ± S.E.M. (n = 10). The statistical significance of differences between rats fed HFD and either SD (#p < 0.001) or HFD supplemented with ERC, EPG or EGG (*p < 0.05) was determined.

As shown in Figure 4A, the serum corticosterone concentration was elevated 2.17-fold in HFD rats compared to SD rats (p < 0.01). The supplementation of HFD with ERC and EPG significantly attenuated the rise of corticosterone level by 27.0 and 30.0%, respectively (p < 0.05), compared to HFD rats, while EGG intake did not significantly influence the index. Adrenal corticosterone levels in the HFD group were 2.15-fold reduced compared to the SD group (Figure 4B, p < 0.001). As shown in Figure 4B, ERC restored the corticosterone content in the adrenal gland by 25.0% (p < 0.05). In contrast, EGG and EPG supplementations did not alter adrenal gland corticosterone levels.

### Effects on serum TNF-α and IL-6

**Figure 5:**
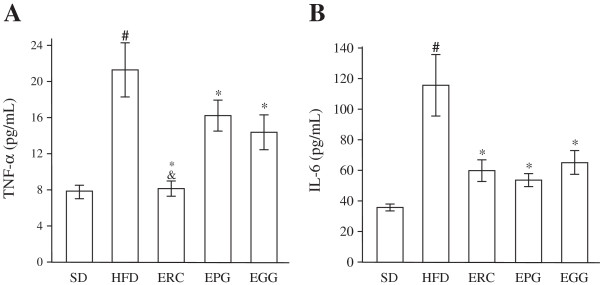
**Concentration of serum TNF-α (A) and IL-6 (B) of rats fed standard diet (SD), high-fat diet (HFD), and HFD supplemented with *Rhaponticum cathamoides *extract (ERC), *Punica granatum *extract (EPG) and *Glycyrrhiza glabra *extract (EGG) (300 mg/kg per day) for 8 weeks.** Data represent the mean ± S.E.M. (n = 10). The statistical significance of differences between rats fed HFD and either SD (#p < 0.001) or HFD supplemented with ERC, EPG or EGG (*p < 0.05), between rats fed HFD supplemented with ERC and either EPG or EGG (&p < 0.001) was determined.

As shown in Figure 5, exposure of rats to an HFD for eight weeks resulted in 2.7- and 3.1-fold increase of TNF-α and IL-6 serum content, respectively, compared to the SD group (p < 0.001). ERC supplementation prevented the HFD-induced elevation of TNF-α serum level, decreasing its value by 61.6% (p < 0.001). In contrast, EGG and EPG reduced the serum TNF-α level to a lesser extent (by 32.0 and 24.0%, respectively, p < 0.05) than ERC (Figure 5A). The ERC group significantly differed from EGG and EPG groups in TNF-α content (p < 0.05). Figure 5B shows that ERC, EPG, and EGG intake resulted in a significant decrease in IL-6 serum level by 48.4, 54.0, and 44.0%, respectively (p < 0.01).

### Effects on liver TG Content, and DNA binding activity of PPARα

**Figure 6:**
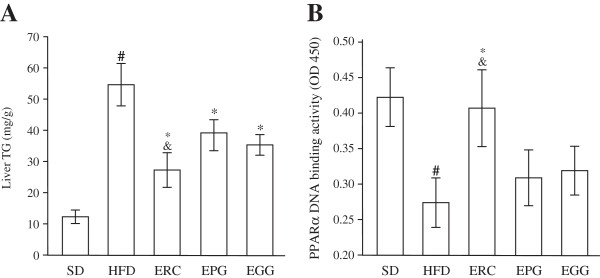
**Liver triacylglycerol (Liver TG) content (A) and PPARα DNA-binding activity (B) of rats fed standard diet (SD), high-fat diet (HFD), and HFD supplemented with *Rhaponticum cathamoides *extract (ERC), *Punica granatum *extract (EPG) and *Glycyrrhiza glabra *extract (EGG) (300 mg/kg per day) for 8 weeks.** Data represent the mean ± S.E.M. (n = 10). The statistical significance of differences between rats fed HFD and either SD (#p < 0.001) or HFD supplemented with ERC, EPG or EGG (*p < 0.05), between rats fed HFD supplemented with ERC and either EPG or EGG (&p < 0.05) was determined.

Liver concentration of TG was significantly higher in HFD rats than in SD rats (p < 0.001). Accumulation of TG was significantly attenuated by supplementation of all three studied herb extracts (Figure 6A). However, ERC was able to reduce liver TG content to a greater extent (by 49.0% compared to the HFD group, p < 0.01) than EPG (by 35.0%, p < 0.01) and EGG (by 29.0%, p < 0.05). TG accumulation was associated with significant decline in DNA binding activity of PPARα in liver of the HFD group compared to the SD group (Figure 6B). Activation of PPARα is known to improve hepatic lipid metabolism. Data presented in Figure 6B confirm a significant recovery of PPARα DNA binding activity in the ERC group (by 48.5%, p < 0.01) compared to the HFD group. In contrast, a minor increase of PPARα DNA binding activity was observed in rats receiving EGG and EPG (p > 0.05).

## Discussion

Feeding of rats on HFD is a useful tool for the induction of metabolic syndrome features including distinctive visceral adiposity, dyslipidemia, impaired glucose tolerance with diabetes type 2, and hepatic steatosis, which are typically associated with human obesity. This rat model of diet-induced obesity is often used to investigate the effects of metabolic syndrome ameliorating agents, including the medicinal plants, as possible sources of new drugs. Various types of extracts and individual compounds derived from *R. carthamoides* have been found to possess a broad spectrum of pharmacological effects [21,24-27]. However, the possibility that extracts and individual compounds derived from *R. carthamoides* may inhibit the manifestation of metabolic syndrome has been studied scarcely, unlike many other herbs such as *G. glabra* and *P. granatum*[13-20]. Our comparative study seems to be the first evidence of the higher potential of the commercial ethanolic ERC extract, enriched in 20-hydroxyecdisone, to reduce the weight of epididymal adipose tissue and serum glucose level, to restore disturbances of serum lipids, corticosterone and inflammatory cytokine content in HFD-fed rats, compared to the commercial ethanolic EGG and EPG extracts, which enriched in glycyrrhizic acid and ellagic acid, respectively, and are believed to have antidiabetic and anti-obesity properties. This study also shows that ERC intake can ameliorate the hepatic steatosis that is associated with an increased expression of hepatic DNA binding activity of PPARα.

Supplementation of ERC, EGG and EPG to an HFD rat (300 mg/kg per day) did not change the body weight of rats in our experiment. However, the epididymal fat weight was significantly reduced by ERC only (Figure 1). In the present study, ERC normalized the plasma glucose level and improved glucose intolerance (Figure 2), decreased LDL-C, TC, TG, and FFA and increased HDL-C content (Table 1) to a significantly greater extent than EGG or EPG. In addition, ERC did not significantly change the systolic pressure of rats (Figure 3). ERC contains a relatively high concentration of phytoecdysteroids, mainly, 20-hydroxyecdysone (2.2% by HPLC data). Diverse extracts and ecdysteroids derived from *R. carthamoides* are widely used in sport medicine as anabolic substances strengthening biosynthesis in slow muscle fibers. The significant decrease of epididymal fat weight without change of body weight and improvement of glucose intolerance observed in rats receiving ERC (containing approximately 6.6 mg/kg per day 20-hydroxyecdysone) are most likely due to the activation of the pentose phosphate pathway and utilization of carbohydrates in protein synthesis stimulated by phytoecdysteroids. This assumption is consistent with the reduced hepatic glucose production and increased adiponectin production, as seen in mice fed with an HFD supplemented with 10 mg/kg per day 20-hydroxyecdisone [28]. Recent trials in ovariectomized rats also showed that modest doses of 20-hydroxyecdysone (18 mg/day per animal) increased muscle mass and reduced visceral fat mass, lowered serum LDL content, raised serum HDL content, and did not elevate TG content [29].

Although the hypocholesterolemic action of phytoecdysteroids has been demonstrated before [30], the molecular mechanisms underlying the effects of ERC in mammals are insufficiently studied. It is known that 20-hydroxyecdisone is the insect steroid hormone, which controls lipid metabolism through a specific receptor complex ecdysone receptor (EcR) [31]. EcRs are orthologs of farnesoid X receptors (FXR) and liver X receptors (LXR) [32], which play critical roles in the regulation of lipid metabolism in mammals. Keeping in mind that the EcR ligand binding domain is similar to the rat FXR and possesses high homology with LXR [33], it may be assumed that these receptors are the pharmacological targets of ERC phytoecdysteroids or their metabolites. Moreover, interaction of LXR with hormone responsive elements in the promoters of PPAR genes may provide for the mutual coordination of expression of these transcription factors.

Our finding of the significant beneficial effect of ERC on hepatic steatosis can be explained by an increase of PPARα DNA binding activity in liver (Figure 6). Mechanisms of this ERC action remain unclear. We can speculate that it is accomplished through direct interaction of phytosterols with FXR, LXR or other transcription factors, or indirectly (by changing blood hormonal levels). In the latter case, promotion of liver PPARα activity may be interconnected with reduction of insulin level. Indeed, insulin is known to strongly downregulate the PPARα mRNA level in rat hepatocytes [34]. Decrease of insulin level by the action of 20-hydroxyecdisone can therefore upregulate PPARα and promote its hepatic activity as observed in the present study. Also, one cannot exclude that demonstrated hypolipidemic effects (decrease of serum TG, serum FFA, and liver TG content) of ERC might be due not only to phytosterols, but also to the individual or synergistic action of flavonoids and other active plant phytochemicals.

Considerable evidence from human studies [35,36] and HFD-fed rodent models [37,38] suggests that increased blood or tissue levels of glucocorticoids and pro-inflammatory cytokines play a critical role in the development of metabolic syndrome. In the present study, we found that ERC exhibited a significant ability to reduce serum corticosterone content and restore its adrenal content (Figure 4), as well as to decrease the HFD-induced serum TNF-α and IL-6 levels (Figure 5), while EGG and EPG exhibited significantly less activity of that kind. Our observations agree with the data obtained in cultured HeLa cells in which ERC efficiently inhibited nuclear factor kappa B (NF-κB) [25] involved in cellular responses to inflammatory stimuli and stress. Interestingly, 20-hydroxyecdisone inhibited NF-κB activation less efficiently than ERC [25], indicating the presence of the other anti-inflammatory compounds in ERC, which are more active, than 20-hydroxyecdisone.

While *R. carthamoides* contains natural and low toxicity compounds [21], ERC elicited excellent outcomes without inducing side effects such as diarrhea, and in the present study, none of the animals died in the course of experiment (data not shown). In this emerging context, there are case reports in the literature that suggest that extensive intake of licorice may cause development of hypokalemia, edema, hypertension, and thrombocytopenia [39]. Ellagic acid (up to 40.0% in studied EPG) could induce a hypercoagulation state in mice, rats, and rabbits [40]. Moreover, the ellagic acid-rich pomegranate extracts have a serious drawback of being unstable in aqueous solutions [41].

Thus, complex action of ERC ethanolic extract exceeds the compensatory effects of EGG and EPG ethanolic extracts upon the metabolic syndrome cluster, and, thus, ERC can be proposed for the use as an efficient therapeutic option for the patients who refuse a low-fat diet for treatment of metabolic syndrome.

## Conclusions

Our findings obtained with an HFD rat model suggest that ERC may prevent and ameliorate the signs of metabolic syndrome as a result of the complex beneficial effects on abdominal obesity, glucose intolerance, dyslipidemia, hepatosis, corticosterone and pro-inflammatory cytokine content with no visible signs or symptoms of toxicity in rats indicating a high margin of safety. The extract of *R. carthamoides* roots exhibited complex beneficial activity, which exceeded that of the commercial ethanolic extracts of *G. glabra* roots and *P. granatum* plant enriched in glycyrrhizic acid and ellagic acid, respectively, with proclaimed antidiabetic and anti-obesity properties. Accordingly, extract of *R. carthamoides* traditionally used mainly as a restorative substance is expanded by data from this study supporting evidence that natural ingredients of *R. carthamoides* are promising candidates for drug and food constituents to prevent or ameliorate the most prevalent manifestation of lifestyle-related cluster diseases caused by an extensively HFD.

## Abbreviations

EcR: Ecdysone receptor; EGG: Extract of *G. glabra*; EPG: Extract of *P. granatum*; ERC: Extract of *R. carthamoides*; FFA: Free fatty acid; FXR: Farnesoid X receptors; G. glabra: *Glycyrrhiza glabra*; HDL-C: High density lipoprotein cholesterol; HFD: High-fat diet; IL-6: Interleukin-6; LDL-C: Low-density lipoprotein cholesterol; LXR: Liver X receptors; P. granatum: *Punica granatum*; NF-κB: Nuclear factor kappa B; PPARα: Peroxisomal proliferator-activated receptor-α; R. carthamoides: *Rhaponticum carthamoides*; SD: Standard diet; TC: Total cholesterol; TG: Triacylglycerols; TNF-α: Tumor necrosis factor-α.

## Competing interests

The authors declare that they no competing interests.

## Authors’ contributions

All authors contributed equally in data acquisition. MD drafted the manuscript and all authors contributed to further writing of the manuscript. All authors read and approved the final manuscript.

## Pre-publication history

The pre-publication history for this paper can be accessed here:

http://www.biomedcentral.com/1472-6882/14/33/prepub
